# Chitin digestibility is dependent on feeding behaviors, which determine acidic chitinase mRNA levels in mammalian and poultry stomachs

**DOI:** 10.1038/s41598-018-19940-8

**Published:** 2018-01-23

**Authors:** Eri Tabata, Akinori Kashimura, Azusa Kikuchi, Hiromasa Masuda, Ryo Miyahara, Yusuke Hiruma, Satoshi Wakita, Misa Ohno, Masayoshi Sakaguchi, Yasusato Sugahara, Vaclav Matoska, Peter O. Bauer, Fumitaka Oyama

**Affiliations:** 1 Department of Chemistry and Life Science, Kogakuin University, Hachioji, Tokyo 192-0015 Japan; 2 Laboratory of Molecular Diagnostics, Department of Clinical Biochemistry, Hematology and Immunology, Homolka Hospital, Roentgenova 37/2, Prague, 150 00 Czech Republic; 3 Bioinova Ltd., Videnska 1083, Prague, 142 20 Czech Republic

## Abstract

Chitin, a polymer of *N*-acetyl-D-glucosamine (GlcNAc), functions as a major structural component in chitin-containing organism including crustaceans, insects and fungi. Recently, we reported that acidic chitinase (Chia) is highly expressed in mouse, chicken and pig stomach tissues and that it can digest chitin in the respective gastrointestinal tracts (GIT). In this study, we focus on major livestock and domestic animals and show that the levels of Chia mRNA in their stomach tissues are governed by the feeding behavior. Chia mRNA levels were significantly lower in the bovine (herbivores) and dog (carnivores) stomach than those in mouse, pig and chicken (omnivores). Consistent with the mRNA levels, Chia protein was very low in bovine stomach. In addition, the chitinolytic activity of *E. coli*-expressed bovine and dog Chia enzymes were moderately but significantly lower compared with those of the omnivorous Chia enzymes. Recombinant bovine and dog Chia enzymes can degrade chitin substrates under the artificial GIT conditions. Furthermore, genomes of some herbivorous animals such as rabbit and guinea pig do not contain functional Chia genes. These results indicate that feeding behavior affects Chia expression levels as well as chitinolytic activity of the enzyme, and determines chitin digestibility in the particular animals.

## Introduction

Chitin, a liner β-1, 4-linked polymer of *N*-acetyl-D-glucosamine (GlcNAc), is the second most abundant polysaccharide in nature and functions as a major structural polymer in many organisms^1^. Chitin has been found in organisms living in a wide range of environments ranging from terrestrial to underwater habitats (insects^2–4^, spiders^5^, fungi^6–9^, protists^10,11^, crabs^12,13^, lobsters^14^, shrimps^15,16^, corals^17^, mollusk^18,19^, polychaetes^20^, diatoms^21,22^ and freshwater and marine sponges^23–25^). It exists in three polymorphs, α^26–28^, β^28^, and γ^29,30^, which differ in the orientation and packing of the chitin molecular chains.

Chitin-containing organisms, in particular insects, have recently become attractive as a potential novel animal feed resource due to their nutritional values, production cost and a low impact on the environment^31–33^.

Chitinases (EC 3.2.1.14; KO 1183) hydrolyze the β-1, 4 glycoside bonds of chitin. Mammals, including mice and humans, do not synthesize chitin but possess two active chitinases, chitotriosidase (Chit1) and acidic chitinase (hereafter referred to as “Chia”; alternative name: acidic mammalian chitinase, AMCase) in their genomes^34,35^. These mammalian chitinases belong to the family 18 of glycoside hydrolases^35–37^.

The levels of Chit1 are significantly upregulated in Gaucher disease, chronic obstructive pulmonary disease (COPD), Alzheimer’s disease, atherothrombosis, diabetes mellitus, cystic fibrosis as well as in smokers^38–44^.

Significant increase in Chia mRNA and protein levels has been detected in an induced asthma mouse model as well as in antigen-induced mouse models of allergic lung inflammation^45,46^. In addition, it has been shown that there are single nucleotide polymorphisms in human Chia, which are associated with asthma^47–49^.

Recently, it has been shown that Chia can function as a protease-resistant major glycosidase under the gastrointestinal conditions in mouse, chicken and pig^50–52^. However, the gene expression and enzymatic activity level in other mammals are still unknown. Here, we report that high Chia mRNA level in stomach is dependent on the feeding behavior of the animals. Chia mRNA expression levels were much lower in bovine (herbivores) and dog (carnivores) than those in mouse, pig and chicken (omnivores) stomachs. Moreover, the chitinolytic activities of recombinant bovine and dog Chia enzymes were slightly but significantly lower when compared with those of mouse, pig and chicken Chia. Thus, feeding behavior seems to be directly linked to the Chia mRNA expression.

## Results

### Chia mRNA level is very low in bovine and dog stomach tissues

Previously, Chia mRNA has been reported to be highly expressed in mouse, chicken and pig stomach tissues^50–52^. To compare the chitinase mRNA levels in other livestock and domestic animals, total RNAs from normal bovine and dog tissues were analyzed using a quantitative real-time PCR (qPCR) assay^50–54^ using a single standard DNA molecule (Supplementary Fig. S1). Pepsinogen^55^ and glyceraldehyde-3-phosphate dehydrogenase (GAPDH)^56^ were used as reference genes.

**Figure 1:**
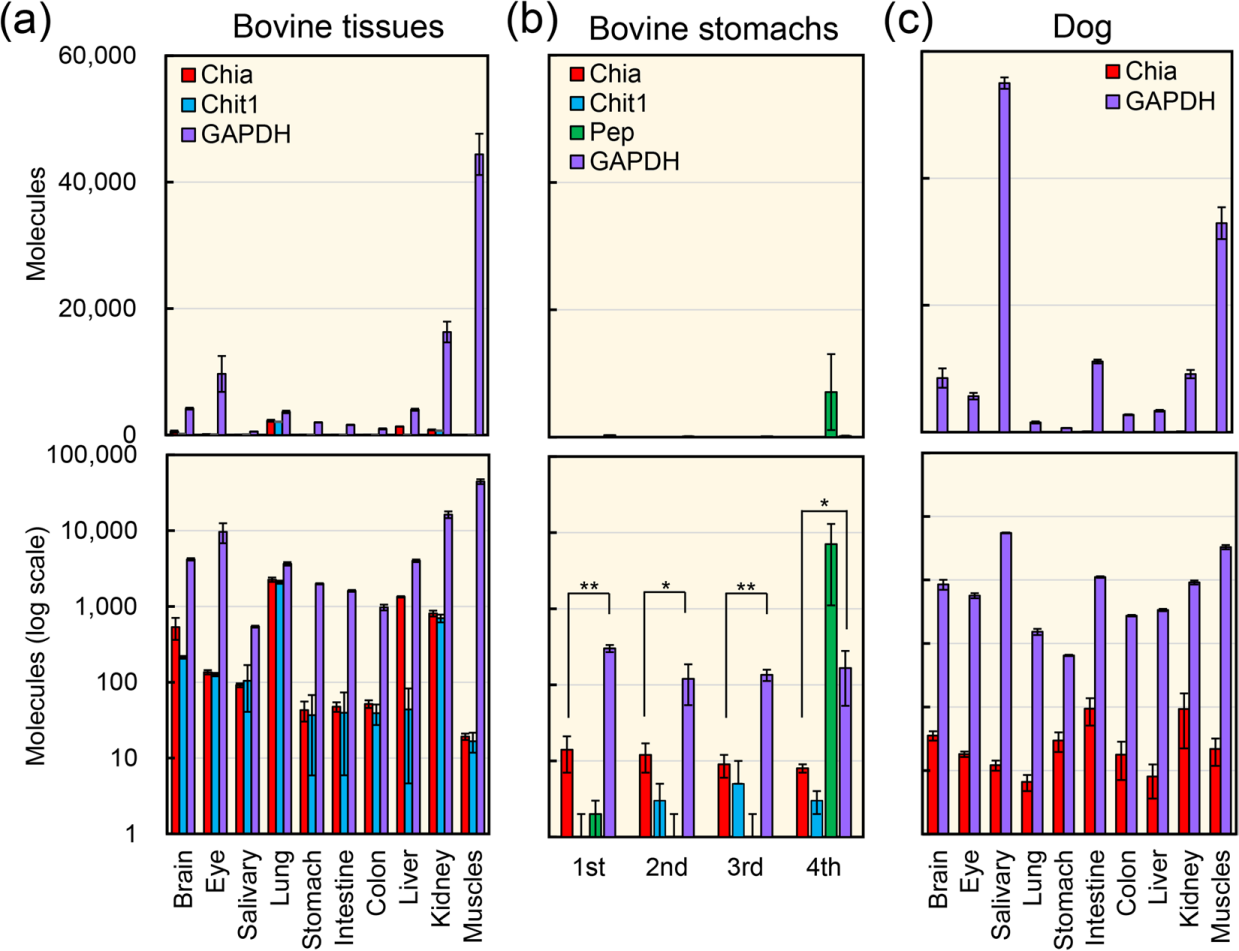
Expression of Chia and Chit1 mRNAs in bovine and dog tissues. The evaluation of Chia and Chit1 mRNA expression in bovine (**a** and **b**) and dog (**c**) tissues using a standard DNA containing genes fragments including Chia, GAPDH, pepsinogen (Pep) and Chit1 of bovine and Chia and GAPDH of dog. Both chitinases were quantified by qPCR using the standard DNA. All values are expressed as molecules per 10 ng of total RNA. All mRNA copy numbers were derived based on the same standard dilutions. The upper panel indicates the actual number, whereas the lower panel shows each value on logarithmic scale. *p < *0.05*, **p < *0.01*. P-values were determined using Student’s t-test.

Bovine Chia mRNA levels were highest in lung and liver (Fig. 1a), which are consistent with a previous report^57^. High levels of Chit1 mRNA were detected in the lung and kidney (Fig. 1a). However, expression of Chia and Chit1 mRNAs were lower than that of GAPDH, a housekeeping gene constitutively expressed in most tissues^56,58,59^.

We also compared the expression levels of the chitinases and reference genes in all four bovine stomachs tissues. The quantitative data are shown in Fig. 1b. Chia mRNA levels were similar in all stomachs, not exceeding those of GAPDH (Fig. 1b). On the other hand, Chit1 mRNA was very low in these tissues (Fig. 1b). Pepsinogen A is an aspartic protease being a major component within the set of gastric enzymes^55^. This protein is also abundantly present in the mouse, chicken and pig stomachs^50–53^. In bovine, pepsinogen A mRNA was predominantly expressed in the fourth stomach (so-called “abomasum”) exceeding GAPDH (Fig. 1b).

Next, gene expression analysis in dog tissues was performed. According to the NCBI genome database, Chit1 gene is not present in the dog genome. Chia mRNA was expressed at relatively high levels in the intestine, kidney, stomach and brain (Fig. 1c). However, it was low in lung and liver (Fig. 1c), where Chia has been reported to be highly expressed in mouse, chicken, pig and human^50–54^. The GAPDH mRNA levels exceeded those of Chia in all examined dog tissues (Fig. 1c).

**Figure 2:**
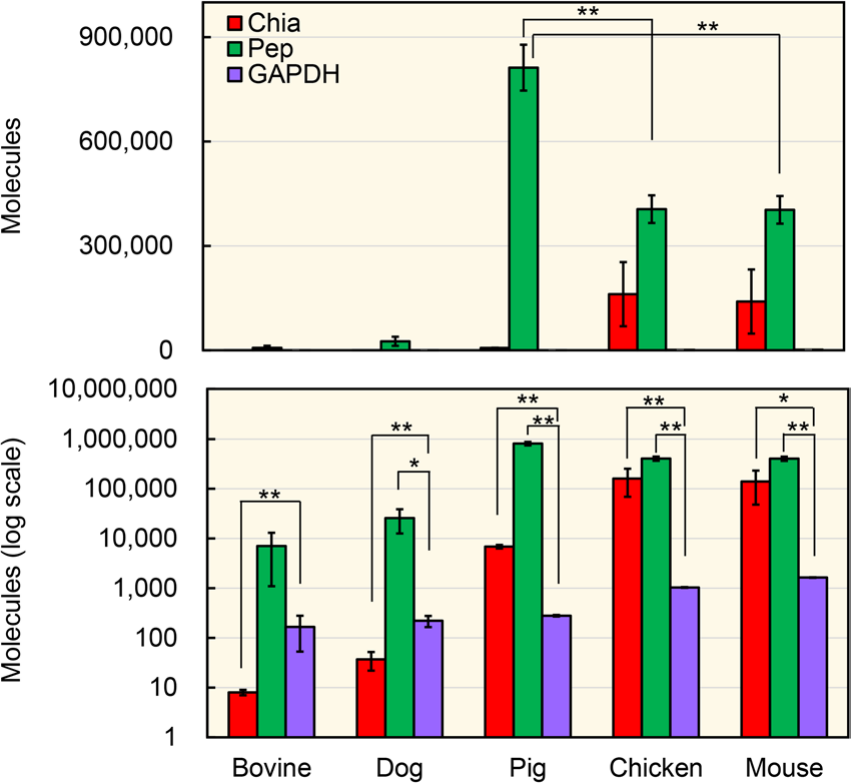
Chia mRNA is highly expressed in omnivores stomach tissue. Expression levels of Chia and gastric genes as well as GAPDH were quantified on the same scale by qPCR using the standard DNA (Supplementary Fig. S1) in bovine, dog, pig, chicken and mouse stomach tissues. Y axis represents molecules per 10 ng of total RNA. Pep, pepsinogen. The upper panel indicates the actual number, whereas the lower panel shows each value on logarithmic scale. Values represent mean ± SD conducted in triplicate. *p < 0.05, **p < *0.01*.

Next, the expression levels of the chitinases and the reference genes using the standard DNA (Supplementary Fig. S1) and cDNAs reverse-transcribed from bovine, dog, pig, chicken and mouse stomach total RNAs were compared (Fig. 2). Bovine and dog stomachs expressed Chia mRNA at low levels, 1/60 and 1/6 of GAPDH, respectively. In contrast, Chia mRNA expression was prominent in mouse and chicken stomachs with levels 86 and 156 times higher than GAPDH, respectively. In pig, Chia mRNA was 25 times higher than GAPDH (Fig. 2). These results indicate that bovine (herbivores) and dog (carnivores) express low amounts, while omnivores (mouse, pig and chicken) express excessive amounts of Chia mRNA.

Pepsinogen mRNA levels exceeded the GAPDH in tested all stomach tissues (Fig. 2). It was very high in the pig stomach tissue and it was almost 3,000 times higher than that of GAPDH. In the bovine and dog stomach tissues, the pepsinogen mRNA levels were 40 and 120 times higher than that of GAPDH, respectively. These results indicate that, when compared to pig, both Chia and pepsinogen mRNAs are being produced in the bovine and dog stomachs at low levels.

### Low protein levels of Chia and pepsinogen in bovine stomach extract

**Figure 3:**
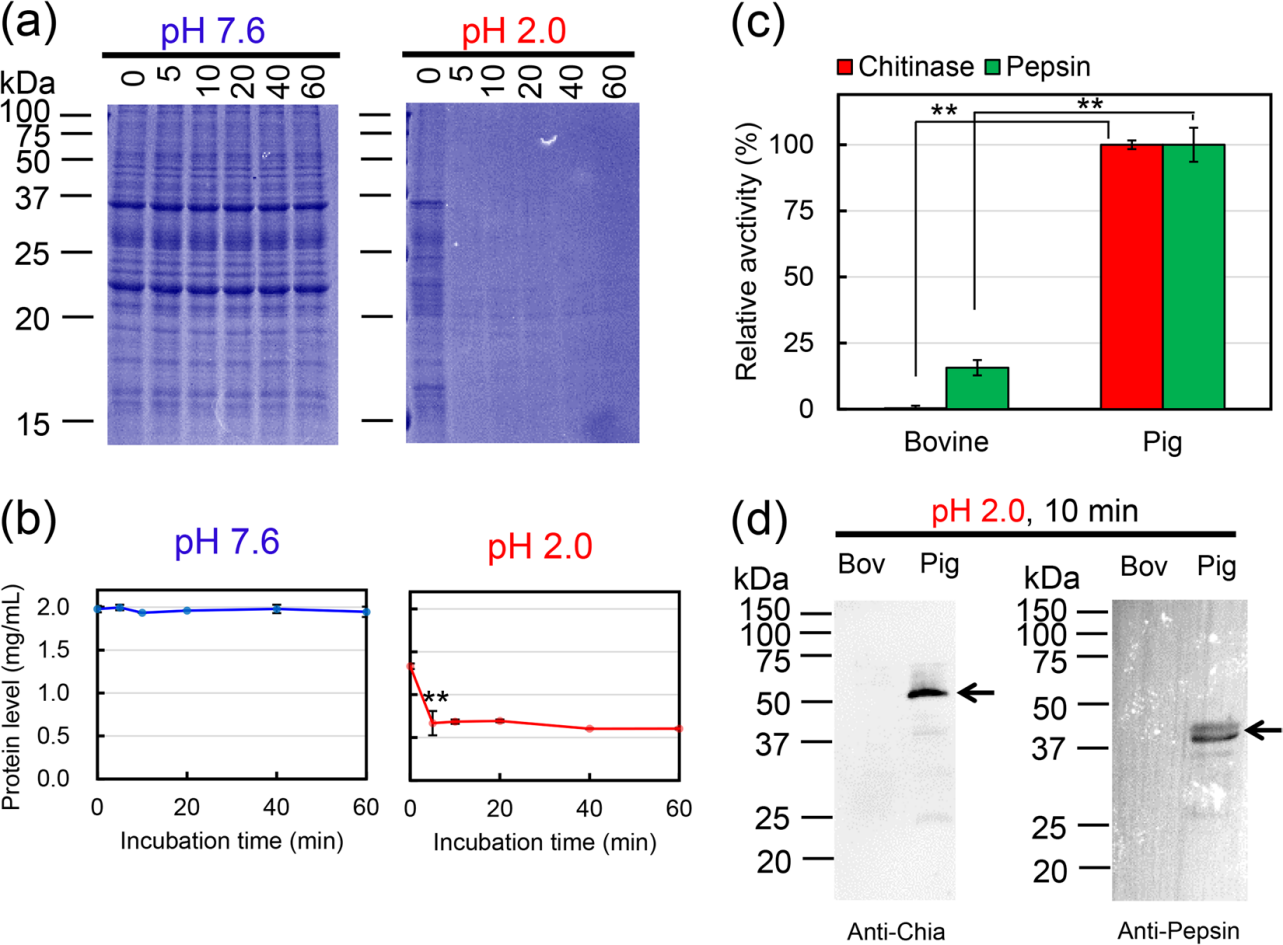
Chia and pepsinogen level in the bovine stomach extract. Soluble protein fraction was prepared from bovine abomasum tissue in the absence of protease inhibitor and incubated at pH 7.6 or pH 2.0 for up to 60 min and analyzed by (**a**) SDS-PAGE, (**b**) total protein levels quantification, (**c**) chitinolytic and protease activity measured in bovine and pig stomach extracts at pH 2.0 as described in the Methods and (**d**) western blot using anti-Chia or anti-pepsin. The images of (**a**) were cropped from original full-length gel images with same exposure time shown in Supplementary Fig. S2a. The images of (**d**) were cropped from original full-length gel images shown in Supplementary Fig. S2b and c, respectively. The image of (**d**) Values in (**b**) and (**c**) represent mean ± SD from a single experiment conducted in triplicate. **p < *0.01*.

We investigated bovine Chia protein and its chitinolytic activity in artificially created bovine stomach environment at pH 2.0 and 37 °C as described previously^50–52^. Soluble protein fraction was prepared from the fourth bovine stomach (abomasum) in the absence of protease inhibitor and incubated at pH 7.6 or pH 2.0 for up to 60 min. The protein fractions were analyzed by SDS-polyacrylamide gel electrophoresis (PAGE), followed by Coomassie Brilliant Blue (CBB) staining (Fig. 3a). At pH 7.6, no changes in the band pattern and intensities were noticed during the 60 min incubation (Fig. 3a,b). In contrast, time-dependent decrease of the soluble proteins with a marked reduction was observed after as early as 5 min of incubation at pH 2.0 (Fig. 3a,b).

To evaluate the protein levels of Chia and pepsin, soluble protein analysis from bovine and pig stomachs (5.0 μg each) after incubation at pH 2.0 for 10 min was performed. The chitinolytic and pepsin activities in pig were significantly higher than those in the bovine stomach (Fig. 3c). In accordance, the Western blot analysis using anti-Chia and anti-pepsin antibodies showed that these enzymes were undetectable after 10 min of incubation at pH 2.0 in the bovine stomach extract while they were still present in the pig stomach extract (Fig. 3d). These results indicate that, consistently with the mRNA levels, Chia as well as pepsin proteins in bovine stomach are very low (Fig. 1b, 2).

### Activity of bovine and dog Chia is lower than that of the omnivorous animals

**Figure 4:**
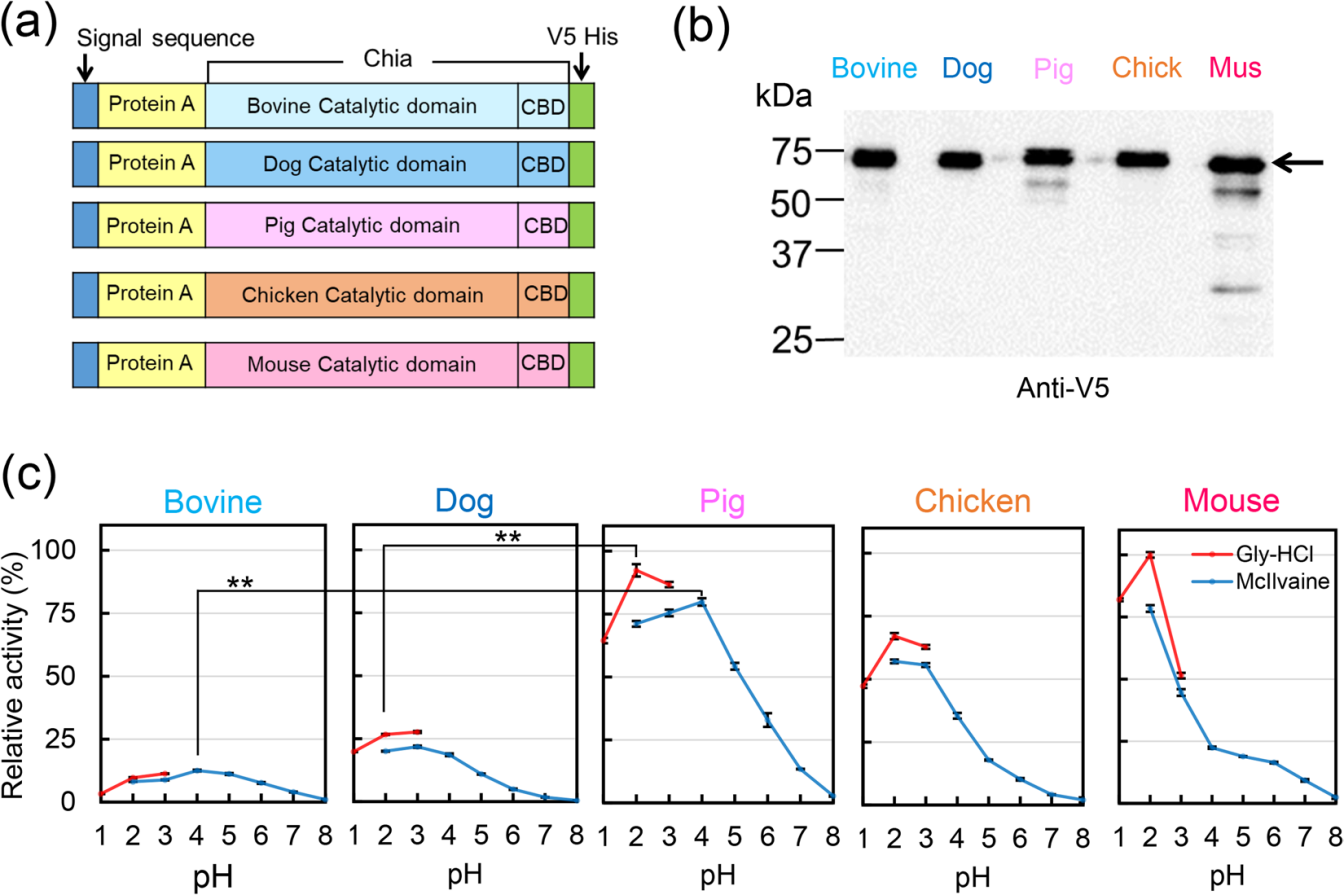
Omnivores Chia activity is higher than that of carnivores and herbivores. (**a**) The schematic representations of the *E. coli*-expressed bovine, dog, pig, chicken and mouse Chia fusion proteins. Chia is a secreted protein with a molecular mass of approximately 50 kDa, which contains an N-terminal catalytic domain (CatD) and a C-terminal chitin-binding domain (CBD). The *E. coli*-produced proteins contain the affinity tail of Protein A at the N-terminus. *E. coli*-recombinant proteins contain V5-His at the C-terminus (Supplementary Fig. S3). (**b**) Western blot analysis of the recombinant proteins using anti-V5 antibody. Arrow highlights the positions of the fusion proteins (Protein A-Chia-V5-His). The image of (**d**) was cropped from original full-length gel image shown in Supplementary Fig. S4a. (**c**) Comparison of the chitinolytic activities of Chia proteins using 4-NP-chitobioside. Error bars represent the mean ± SD from a single experiment conducted in triplicate. **p < *0.01*.

To determine the chitinolytic activity of Chia, bovine, dog, pig, chicken and mouse enzymes were expressed in *E. coli* as fusion proteins with truncated form of *Staphylococcus aureus* Protein A and V5-His tag^49,60^ as described in the Methods (Fig. 4a,b; Supplementary Fig. S3). 4-nitrophenyl *N,N′*-diacetyl-β-D-chitobioside (4-NP-chitobioside) was used as a substrate.

Recombinant mouse and chicken Chia had the highest activity at pH 2.0 and the pig enzyme at pH 2.0–4.0 (Fig. 4c), in accordance with our previous reports^49–52,60,61^. Dog Chia had optimal activity at pH 2.0 while the bovine enzyme is most active at around pH 4.0 (Fig. 4c). However, both showed lower chitinolytic activity as compared to the mouse, chicken and pig Chia with activity levels at 1/6 (bovine) and 1/4 (dog) of that of the pig Chia at pH 4.0 and at pH 2.0, respectively (Fig. 4c).

### Recombinant bovine and dog Chia proteins can degrade chitin substrates under the artificial GIT conditions

**Figure 5:**
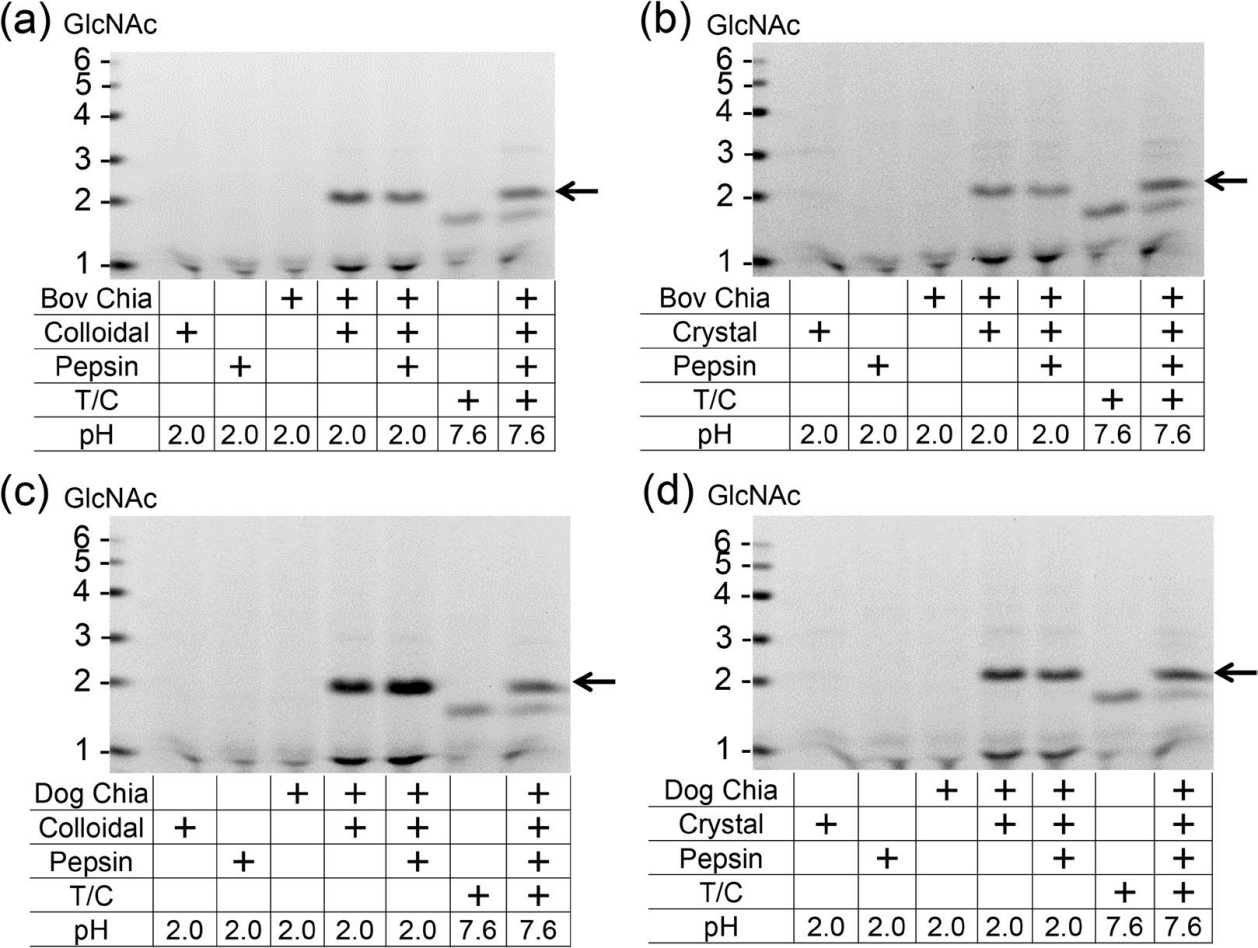
Chitin substrates and chitin-containing organisms are degraded by recombinant Chia enzymes. Recombinant bovine and dog Chia proteins were incubated at 37 °C for 16 hours under GIT-like environment in the presence of pepsin or trypsin/chymotrypsin. Degradation products generated by incubation of (**a** and **c**) colloidal or (**b** and **d**) crystalline chitin in the GIT-mimicking conditions were analyzed by FACE. Arrow indicates the positions of (GlcNAc)_2_. Recombinant bovine and dog Chia proteins can degrade chitin substrates under the artificial GIT conditions. The images of (**a**–**d**) were cropped from original full-length gel images with same exposure time shown in Supplementary Fig. S5.

Even though the recombinant bovine and dog Chia proteins have low chitinolytic activity, we investigated, whether they do possess the ability to degrade polymeric chitin under gastrointestinal tract (GIT) conditions. Colloidal and crystalline chitin were incubated with bovine (Fig. 5a,b) and dog (Fig. 5c,d) fusion proteins under conditions mimicking GIT environment. The enzymes released mainly (GlcNAc)_2_ from the colloidal (Fig. 5a,c) as well as the crystalline (Fig. 5b,d) substrate. These results imply that both bovine and dog Chia are able to degrade chitin in the GIT.

### Functional and pseudo Chia genes adapted by the feeding behavior

**Figure 6:**
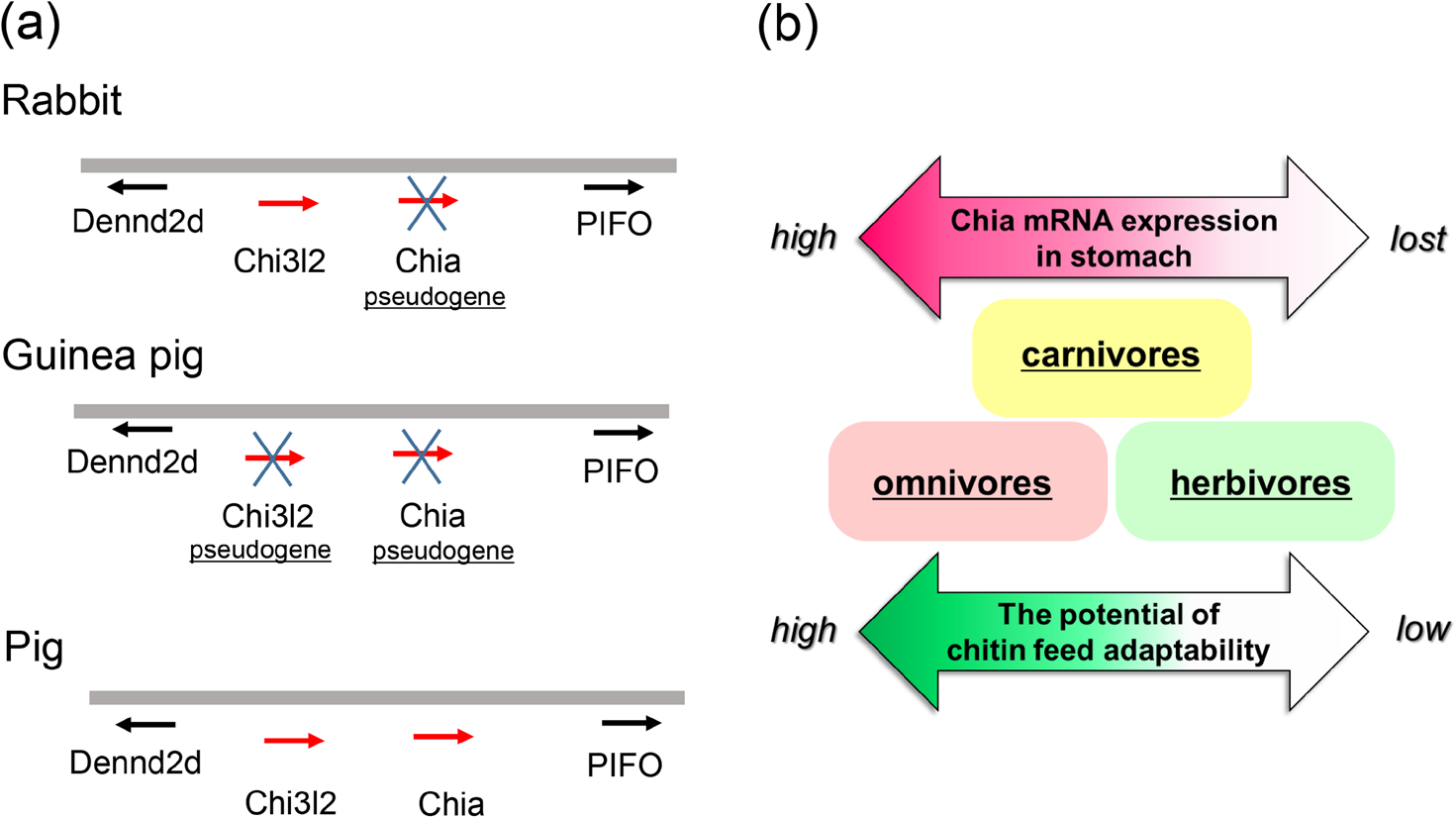
The Chia gene evolution affected by the feeding behavior, which may determine chitin digestibility. (**a**) Schematic representation of the Chia genes as well as neighboring marker genes. Rabbit and guinea pig (herbivorous animals), do not have Chia gene in their genomes. Chia-like regions can be found between Dennd2d and PIFO. The vestigial genes lacking protein-coding abilities were found in the regions. (**b**) The relationship between feeding behavior and chitin digestibility in major livestock as well as laboratory and domestic animals based on Chia transcriptional levels in the stomach, chitinolytic activity and pseudogenization.

As shown above, Chia mRNA level was significantly lower in the bovine (herbivore) in comparison to omnivores (Fig. 2). The NCBI Gene search showed that genomes of some herbivorous animals, such as rabbit and guinea pig, do not contain Chia genes (Fig. 6a). To further examine whether rabbit and guinea pig possess sequences similar to Chia genes in their genomes, we searched the regions between DENN/MADD Domain Containing 2D (DENND2D) and Pitchfork (PIFO) genes by NCBI blast search. These genes are conserved in mouse, rabbit, guinea pig, pig and human and are used as landmark genes in the vicinity of the Chia gene. DENND2D is a candidate tumor suppressor^62,63^, while PIFO regulates cilia disassembly^64^. Although we found vestigial Chia genes in both rabbit and guinea pig, they lack protein-coding abilities resulting from change to inactive pseudogenes (Fig. 6a).

Based on the Chia transcription level and chitinolytic activity in stomach as well as pseudogenization, we summarized the relationship between the feeding behavior and chitin digestibility in major livestock as well as laboratory and domestic animals as shown in Fig. 6b. The chitin adaptability seems to be higher in omnivores with sufficient levels of Chia mRNAs and proteins in their stomach tissues as compared to carnivores and herbivores with low levels and activity of Chia.

## Discussion

In previous reports, we have shown that mouse, chicken and pig stomach tissues express high levels of Chia mRNA and their translation products can degrade chitin substrates into (GlcNAc)_2_ under GIT conditions^50–52^. These results led to a speculation that all animals express large amounts of Chia enzyme in their stomach tissues. Here we reported that this observation is not reflected in all animals and is restricted to the omnivores. The Chia mRNA level in the stomach is strongly related to the feeding behaviors. Also, its chitinolytic activity is correlated to Chia transcription levels. Furthermore, some herbivores such as rabbit and guinea pig do not have functional Chia genes in their genomes. These results imply that feeding behaviors determine the Chia mRNA levels and its specific activity as well as Chia gene conservation during the evolution.

Bovine is a ruminant with four stomachs. Chia mRNA was expressed at low levels in all stomachs when compared with other animals. Although dog is a monogastric animal, Chia mRNA level was also very low in the stomach as well as in other tested tissues. Since dogs do not possess Chit1 gene in the genome, chitinases might to play different roles than in other mammals.

Pepsin has been detected in variety of animal stomach tissues and studied extensively^55,65^. As far as we know, comparison of their levels among multiple mammalian species has never been reported. As shown in Fig. 2, pepsinogen is a major transcript exceeding GAPDH in pig, chicken and mouse stomach tissues. However, the expression of pepsinogen in bovine and dog stomachs was much lower than in other animals (Fig. 2). Based on these results, we suggest that expression of pepsinogen as well as Chia may be controlled by their feeding behavior in the stomach.

Chitin digestibility can be estimated to some extent by Chia gene expression level in stomach as well as its chitinolytic activity. Our data showed that specific activity of Chia is as follows: mouse: chicken: pig: bovine: dog = 1.0: 0.7: 0.9: 0.25: 0.1. When those mRNA levels (number of molecules) are multiplied by its specific activities, the ratio is roughly: 140,000: 113,000: 6,000: 10: 1. These results clearly indicate that there are substantial differences in Chia mRNA levels and chitinase activities between omnivores, herbivores and carnivores stomach tissues and that chitin-digestibility is primarily determined by Chia mRNA levels.

In this report, we also compared specific activities of recombinant mouse, chicken, pig, bovine and dog Chia proteins. Although the primary sequences share about 70–79% identity, the chitinolytic activities of mouse, chicken and pig Chia were 4-to-10 times higher than those of bovine and dog enzymes. These results suggest that several non-conserved amino acid residues may influence the Chia activity and pH dependency. Furthermore, only the bovine Chia’s chitinolytic activity was highest at pH 4.0 (other species at pH 2.0). These results indicate that herbivorous stomach may have been adapted to express Chia with optimal activity in the less acidic condition.

Sequence changes and expression pattern of gene often determine the response of the organism to environmental stimuli^66^. For example, ruminant-like species have remarkably high concentrations of lysozyme c (EC 3.2.1.17) in the mucosa of the true stomach with differences in the time-, pH- and ionic strength-dependence of the rate of bacterial lysis when compared to conventional mammalian lysozymes c^67^. Similarly, the umami taste receptor gene, Tas1r1, has been shown to become a pseudogene in giant panda during a dietary switch to bamboo due to the relaxation of the functional constraint and ORF-disrupting substitutions^68^. The gene expression and it enzymatic activity of human Chia is affected by an insertion in the 5′ UTR^69^, promoter polymorphisms^47^ and nonsynonymous substitutions^48,49^. This knowledge allowed us to speculate that changing feeding behavior in some herbivores and carnivores had a major effect on the conservation of coding or non-coding region of the Chia genes.

Organisms containing chitin have been investigated due to their potential as alternative animal diets^32,70–73^. In pilot trials, shrimp shell chitin and dried mealworm (*Tenebrio molitor*) larvae were added to corn-based feeds for chicken and porcine diets, respectively^74,75^. Both studies showed that such supplementation is safe with effect on survival rate, growth performance, nutrient digestibility and blood profiles. A recent study reported that certain insect-eating primates underwent similar physiological adaptation as observed in mouse, chicken and pig as well as insectivorous bats^76^ as for the Chia enzymes utilization for chitin digestion in the insect exoskeletons^77^.

In this report, we show that, comparing to carnivores and herbivores, omnivores possess high ability of chitin digestion in their GIT. Before introduction of chitin-containing organisms to animal feeds, further research is needed on nutritive quality and safety evaluation of the potential by-products from such materials^72,78,79^.

Bovine stomachs contain high levels of cellulases (major digestive enzyme in herbivores), mainly produced by microorganisms^80,81^. Bovines often ingest insects abundantly present in their diet, e.g. with grass. Since the levels of Chia mRNA in the bovine stomach tissues are very low, it is possible that GIT bacteria supplement the chitinases, similarly to cellulase. Bovine GIT contains billions of bacteria in symbiosis^82^. Thus, bacterial chitinases may indeed play an important role in chitin digestion in bovines and possibly dogs. This hypothesis warrants further scrutiny.

Ingestion of insects by dogs is also not an uncommon event and chitin is usually well-tolerated. However, health issues can be caused by overfeeding. In “chitin overdose” cases in dogs or bovines, a mixture of lyophilized (recombinant or natural Chia from pig or chicken) chitinases or a probiotic treatment^83,84^ by chitinase-producing microbes could represent a potential treatment. Using such agents, chitin-containing organisms could potentially be utilized for non-omnivorous animals’ diets.

## Methods

### Bovine, pig and chicken stomach tissues

Frozen bovine, pig and chicken stomach tissues were purchased from Funakoshi Co., Ltd (Tokyo, Japan). The tissues were dissected and quickly frozen on dry ice and kept at −80 °C.

### Total RNA and cDNA preparation

The Bovine Total RNA Panel and the Dog Total RNA Panel were purchased from Zyagen (San Diego, CA, USA) to examine the distribution of transcripts in various tissues. Mouse Total RNA Master Panel and Pig Total RNA panel were also purchased from Takara Bio USA, Inc. (Mountain View, CA, USA) and Zyagen, respectively. Mouse and pig stomach total RNAs from the panels were used to synthesize cDNAs for analyzing the level of Chia mRNA. In addition, total RNA was isolated from the frozen bovine or chicken stomach tissue using TRIzol Reagent (Thermo Fisher Scientific, Waltham, MA, USA) per manufacturer’s instructions. Total RNAs were reverse-transcribed into cDNA essentially as described previously^50–52^.

### Selection of primer pairs for qPCR

Primers for qPCR were designed using PrimerQuest Input (Integrated DNA Technologies, Coralville, IA, USA) and evaluated their suitability based on whether they gave single products, as reflected by a single melting temperature (Tm). PCR reactions (final volume 13 µL) contained 2× SYBR Green Master Mix (Brilliant II SYBR Green QPCR Master Mix, Agilent, Santa Clara, CA, USA), 2.7 ng of bovine or dog cDNA or appropriate amount of the external standards (see below) and 2.5 pmol of primers for Chia, Chit1, pepsinogen and GAPDH. The PCR reactions were performed using Mx3005P QPCR System (Agilent) as follows: an initial denaturation and polymerase activation step for 10 min at 95 °C, followed by 40 cycles of denaturation at 95 °C for 30 sec, annealing at 55 °C for 30 sec and polymerization at 72 °C for 10 sec. Melting curves were generated after amplification. The primers’ sequences are listed in Supplementary Table S1.

### Construction of the standard DNA and qPCR

Construction of the 18 genes standard DNA containing the genes of major proteins in stomach was described previously^50–52^. The standard DNA (Supplementary Fig. S1) was synthesized and inserted into pTAKN-2 vector by Eurofins Genomics (Tokyo, Japan). The standard DNA was prepared by PCR from the plasmid DNA using the forward primer 5′- GCTGCTGGTATCTCCAACAT -3′ and the revers primer 5′- TGGGCGTGGCTCAGGTAT -3′ and used for qPCR. qPCR was performed using standard DNA and cDNAs reverse-transcribed from total RNAs from animals essentially as described previously^50–52^.

### Bovine stomach extract preparation

Soluble fraction was prepared from bovine fourth stomach tissues (0.2 g) by homogenization followed by centrifugation at 15,000 g for 10 min at 4 °C^50–52^. The supernatants were used as the stomach extract and pre-incubated at 37 °C for 0, 10, 20, 40 or 60 min at pH 7.6 or pH 2.0. After incubation at pH 2.0 and 37 °C, the solutions were neutralized. Total protein levels quantification was carried as described previously^50,51^.

### Pig stomach extract preparation

Soluble fraction was prepared from pig stomach tissues as described previously^52^. After incubation at pH 2.0 and 37 °C for 10 min, the solutions were neutralized. Protein concentration was determined as described above.

### SDS-polyacrylamide gel electrophoresis and western blot

The obtained protein fractions were analyzed using standard SDS-polyacrylamide gel electrophoresis (PAGE), followed by CBB staining. Western blot analysis was performed using polyclonal anti-mouse C terminal Chia^50,54^ or polyclonal goat anti-pepsin^52^, as described previously^50,52^.

### Chitinase enzymatic assays

Chitinase enzyme activity was determined with 4-nitrophenyl-*N, N*′-diacetylchitobioside (Sigma-Aldrich, St. Louis, MO, USA) as a substrate in McIlvaine’s buffer (0.1 M citric acid and 0.2 M Na_2_HPO_4_; pH 2.0 to pH 8.0) or 0.1 M Gly-HCl buffer (pH 1.0 to pH 3.0) at 37 °C for 1 hour as described previously^49,54^. Chia unit definition was also reported previously^60^.

### Pepsin enzymatic assays

Pepsin activity was measured using hemoglobin from bovine blood (Sigma-Aldrich) as the substrate as described previously^50^.

### *E. coli* expression vectors

Coding regions of mature form of bovine, dog, pig and chicken Chia cDNAs were amplified from the corresponding animal’s cDNAs by PCR using KOD Plus DNA polymerase (Toyobo Co., Ltd, Osaka, Japan) and oligonucleotide primers (Eurofins Genomics) anchored with the restriction sites for EcoRI and XhoI (Supplementary Table S2) as described previously^60^. Amplified cDNA was digested with EcoRI and XhoI and cloned into the same sites of the pEZZ18/pre-Protein A-Chia-V5-His^60^. The entire nucleotide sequence of the resulting plasmid DNA (pEZZ18/Chia/V5-His) was confirmed by sequencing (Eurofins Genomics). The pEZZ18/pre-Protein A-mouse Chia-V5-His was prepared as described previously^60^. Expression of these plasmid DNA in *E. coli* cells led to the production of the mature Protein A-Chia-V5-His (Fig. 4a; Supplementary Fig. S3).

### Preparation of the recombinant Chia proteins expressed in *E. coli*

Using the plasmid DNAs (the pEZZ18/pre-Protein A-Chia-V5-His), *E. coli* BL21 (DE3) (Merck Millipore, Tokyo, Japan) was transformed to express pre-Protein A-Chia-V5-His proteins. Transformed *E. coli* BL21 (DE3) strains were grown in 1.5 L LB medium containing 100 µg/mL ampicillin at 37 °C for 18 h. Cells were harvested by centrifugation at 7,000 *g* for 20 min at 4 °C. The recombinant protein was prepared from *E. coli* and purified by IgG Sepharose (GE Healthcare, Piscataway, NJ, USA) chromatography as described previously^49,60^. The protein-containing fractions were desalted using PD MidiTrap G-25 (GE Healthcare) equilibrated with the TS buffer [20 mM Tris-HCl (pH 7.6), 150 mM NaCl and a protease inhibitor (Complete, Roche, Basel, Switzerland)]. Protein A-mouse Chia-V5-His was also prepared as described previously^60^. The recombinant products were detected by Western blot using anti-V5-HRP monoclonal antibody (Thermo Fisher Scientific).

### Degradation of colloidal and crystalline chitin substrates

Colloidal chitin and crystalline chitin were prepared from shrimp shell α-chitin (Sigma-Aldrich) and used as substrates to determine the chitinase activity. The degree of deacetylation (DD) of chitin was determined by elemental analysis. The elemental analysis of chitin was performed at the Analytical Center of the Tokyo University of Pharmacy and Life Sciences. Shrimp shell chitin (2.1% of DD) was powdered in a Wiley mill (Thomas Scientific, Swedesboro, NJ, USA) to 250 μm particle size and used as crystalline chitin. Also, it was incubated in concentrated HCl at 40 °C for 30 min and following filtration using a fused-in fritted glass disc (SIBATA SCIENTIFIC TECHNOLOGY LTD, Saitama, Japan), washed extensively with water to attain neutral pH and used as colloidal chitin. All enzymatic reactions using colloidal chitin (at a final concentration of 1 mg/mL) or crystalline chitin (1 mg/reaction) as substrates were incubated in a volume of 50 µL containing recombinant bovine (0.01 mU) or dog Chia (0.03 mU) in the presence of pepsin (0.6 µg) or trypsin/chymotrypsin (0.6 µg) as described previously^52^. *N*-acetyl chitooligoaccharides (Seikagaku Corporation, Tokyo, Japan) were used as a standard.

### Statistical analysis

Biochemical data were compared by Student’s t-test.

### Data availability

The datasets generated and/or analyzed during the current study are available from the corresponding author on reasonable request.

## Electronic supplementary material

Supporting Information
