# Supplementary material for: Chitin digestibility is dependent on feeding behaviors, which determine acidic chitinase mRNA levels in mammalian and poultry stomachs
Source: Sci Rep. 2018 Jan 23;8:1461. doi: 10.1038/s41598-018-19940-8 (PMC5780506; doi:10.1038/s41598-018-19940-8)
Supplement: Supplementary file 1 — Supporting Information [file 41598_2018_19940_MOESM1_ESM.pdf]

Supplementary Information

Chitin digestibility is dependent on feeding behaviors, which determine acidic chitinase mRNA levels in mammalian and poultry stomachs

Eri Tabata[1], Akinori Kashimura[1], Azusa Kikuchi[1], Hiromasa Masuda[1], Ryo Miyahara[1], Yusuke Hiruma[1], Satoshi Wakita[1], Misa Ohno[1], Masayoshi Sakaguchi[1], Yasusato Sugahara[1], Vaclav Matoska[2], Peter O. Bauer[2, 3], Fumitaka Oyama[1, *]

[1]Department of Chemistry and Life Science, Kogakuin University, Hachioji, Tokyo 192-0015, Japan, [2]Laboratory of Molecular Diagnostics, Department of Clinical Biochemistry, Hematology and Immunology, Homolka Hospital, Roentgenova 37/2, Prague 150 00, Czech Republic, [3]Bioinova Ltd., Videnska 1083, Prague 142 20, Czech Republic

(a)

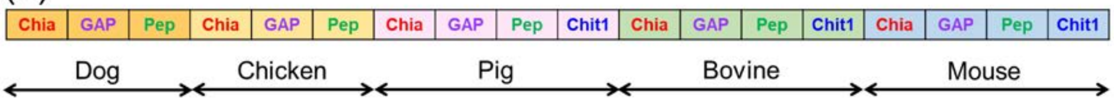

(b)

GCTGCTGGTATCTCCAACATCCAGTCTGGATATGACATCCCCCAGCTGTCCCAGTACCTGGACTACATCCATGTCATGACCTAT
GACTTCCATGCCTCCTGGAACATCATCCCTGCTTCCACTGGCGCTGCCAAGGCTGTGGGCAAGGTCATCCCTGAGCTGAACGGG
AAGCTCACTGGCATGGCCTTCCGTGTCCCCACCCCCAATGTATCAGTTGTGGATCTGACCTGGCAAACCTATACACTGGCCTAT
GGTTTTGGGAGCCTGACTGTGCTCCTGGGATATGACACTGTGACTGTTCAGAACATTGTCATCCATAACCAGCTATTTGGCATG
AGTGAGAATGAACCCAACTACCCATGCTGGACATTGACTGGGAATACCCTGGATCAAAGGGCAGCCCTTCTCAGGACAAAGGTC
TCTTCACCGTCCTTGTTCAGCAACGGATTTGGCCGTATTGGCCGCCTGGTCACCAGGGCTGCCGTCCTCTCTGGCAAAGTCCAA
GTGGTGGCCATCAATGATCCCTTCATCGATCTGAACTGTGGGTGCCCTCTATCTATTGCAAAAGCTCGGCCTGCAGCAACCACA
AACGCTTTGACCCCTCCAAGTCCTCAACCTACGTGAGCACCAACGAAACCGTCTACATCGCCTACCCTGTACTACCTGGCCTTC
TACGTGGTGATGACTGGGATCTTCGCCCTCTCCATATACTCCCTAATGAGGACGGTCAATCCCTACGAGCCAGATTACTGACTT
CACAGGCACTTTCTGCAACCAGGGCAAGTTCCCCCTGATCAACACCCTGAAGGATGCTCTTGGCCTGAATAGCACAAGTTGCAC
CGGTGACACTCACTCTTCCACTTTTGATGCTGGGGCTGGCATTGCCCTCAACGACCACTTCGTCAAGCTCATTTCCTGGTACGA
CAATGAATTTGGCTACAGCAACAGGGGCGGCATCGATTCTTCTTACTACACAGGAAGCCTGAACTGGGTGCCTGTTTCCGTCGA
GGGTTACTGGCAGATTACCTTGGACAGCATCACCCTGGGTTCACTGACTACTTCACGGCCATGGCCCAGGAGGGCTGGTTCCCA
CTGCTGTGTGTGGGGCTGCGGCCACAGTGGGAGAACCACCACCTACAAGACGTCAACTCAGCCATCAGGTTTCTGCGCAAATAT
GGCTTTGATGGCCTTGACCTTGCACTCACCTGATCTATGCCTTCGCTGGGATGAGTAACAGTGAGATCACCACCATTGAATGGA
ATGATGTGGCTCTCTAGATGCTGGTGCTGAGTATGTGGTGGAGTCCACTGGGGTCTTCACTACCATGGAGAAGGCTGGGGCTCA
CTTGAAGGGTGGCGCCAAGAGGGTCATCATCTCTGCACCTTCTGCGTCAAGATCCCACTCGTCAAAAAGAAGTCCTTGAGACAG
AATCTGATCGAGAATGGCAAGCTGAAGGAATTCATGAGGACACACAAATACAACCTGGGCAGCAGCATATGAGAAGGCTGAGAG
TGACATCATGCACTTGCGCCCACGGAACCCAAAGCGGGACCGACTGGTCAACGAGCCCCTGGCTGCCTACTCCTACTTCCAGAT
CGGTGGCCTTTCTGATTGCCATTTCTTTTCTTTGCCCCGACTGGCCAGGCTCTGCTGCAAAACTGGTCTGCTACTTCACTAACT
GGGTCCAGTACAGAGAGGGGTCTGCTTGCTTCTTACCCAAGGATGTGGACTTTTGGCAGTGCATCAATGGAATCACATACCAGC
AGCATTGTCAAGCAGGGCTTGTTTTTGATACCAGCTGTAATTGCTGCTGTGTCCGTCGTGGATCTGACGTGCCGCCTGGAGAAA
CCTGCCAAGTATGATGACATCAAGAAGGTGGTGAAGCAGGTGCCAAGGCATTGTAGACACAGGCACCTCTCTGCTCGTCATGCC
TGCCCAGTACCTGAATGAACTTCTGCAGACCATAGGAGCGGCAGGAACTAAATCTTCCATCCGAGACTCCAAGGAGCCCAGAAC
AGATAATACCTGAGCCACGCCCA

Figure S1. Single standard DNA molecule used for qPCR. (a) Schematic representation of the standard DNA molecule. (b) Nucleotide sequence of the single standard DNA. The single standard DNA, 2,039 bases long, contained cDNA fragments of Chia and Chit1 as well as reference genes [pepsinogen (Pep) and GAPDH (GAP)] of animals shown in (a). Primers for qPCR in each target DNA region are shown in underline.

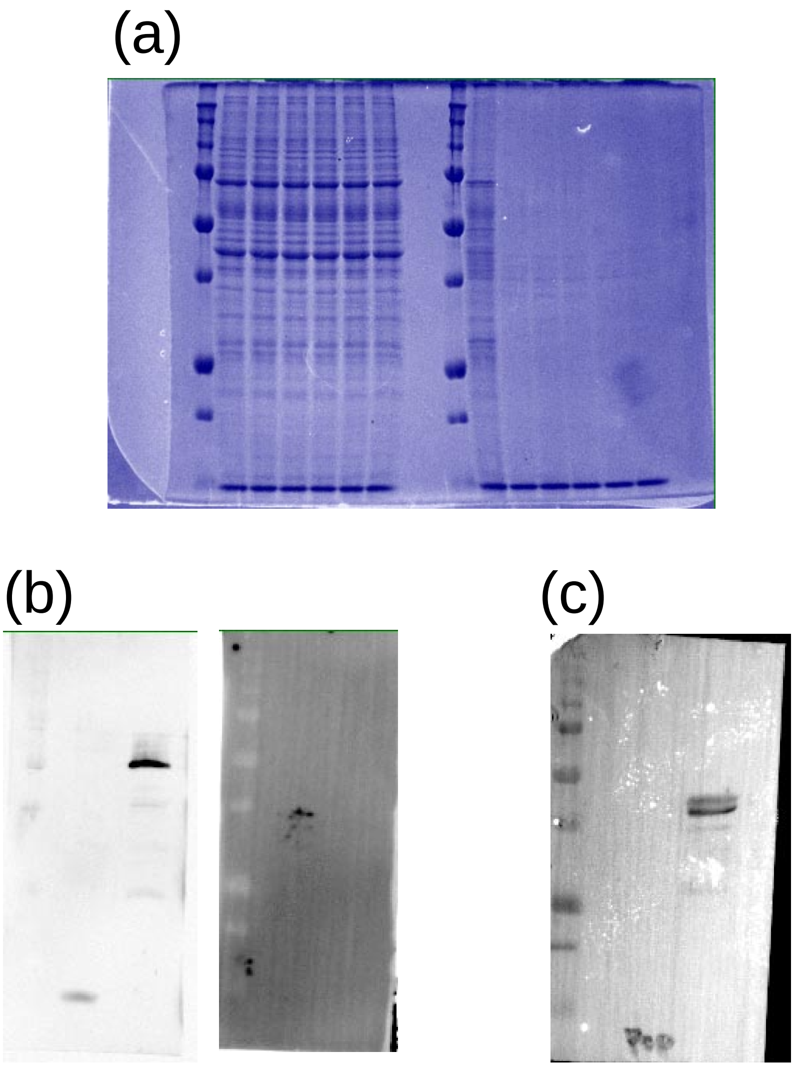

Figure S2. Full-length gel image shown in Fig. 3a. (a) Full-length gel images of SDS-PAGE and CBB staining. (b) Western blotting using anti-Chia (left) and its molecular weight markers (right). (c) Western blotting using anti-pepsin and molecular weight markers.

A. Protein A-bovine-Chia-V5-His
608 amino acids   67,609 dalton

```
AQHDEAVDNKFNKEQQNAFYEILHLPNLNEEQRNAFIQSLKDDPSQSANLLAEAKKLND
AQAPKVDNKFNKEQQNAFYEILHLPNLNEEQRNAFIQSLKDDPSQSANLLAEAKKLNDA
QAPKVDANSYQLVCYFSNWAQYRPGLGSFKPDNIDPCLCTHLIYAFAGMSNSEITTIEW
NDVALYSSFNDLKKKNSQLKILLAIGGWNFGTAPFTAMVATPENRKTFISSVIKFLHQY
GFDGLDFDWEYPGSRGSPSQDKHLFTVLVQETREAFEQEAKQTNKPRLLVTAAVAAGIS
NIQAGYEIPQLSQYLDFIHVMTYDFHGSWEGYTGENSPLYKYPTDTGSNTYLNVEYAMN
YWKKNGAPAEKLIIGFPAYGHNFILRDASNNGIGAPTSGAGPAGPYTREAGFWAYYEIC
AFLKDGATEAWDDSQNVPYAYKGTEWVGYDNVNSFRIKAQWLKENNFGGAMVWAIDLDD
FTGTFCNQGKFPLINTLKDALGLKSAPCNASTQSSEPNSSPGNESGSGNKSSSSEGRGY
CAGKADGLYPVADNRNAFWNCVNGITYKQNCLTGLVFDTSCHCCNWAARGHPFEGKPIP
NPLLGLDSTRTGHHHHHH
```

B. Protein A-dog-Chia-V5-His
636 amino acids   68,443 dalton

```
AQHDEAVDNKFNKEQQNAFYEILHLPNLNEEQRNAFIQSLKDDPSQSANLLAEAKKLND
AQAPKVDNKFNKEQQNAFYEILHLPNLNEEQRNAFIQSLKDDPSQSANLLAEAKKLNDA
QAPKVDANSYQLTCYFTNWAQYRPGLGSFKPDDIDPCLCTHLIYAFAGMKNNEITTIEW
DDVTFYQAFNGLKNKNSQLKTLLAIGGWNFGTAPFTAMVSSPENRQTFIASVIKFLRQY
EFDGLDFDWEYPGSRGSPPQDKHLFTVLVQEMREAFEQEAQQINKPRLMITAAVAAGIS
NIQSGYDIPQLSQYLDYIHVMTYDFHASWEGYTGENSPLYKYPSDTGSNAYLNVDYVMN
YWMDNGAPAEKLIVGFPAYGHTFILSDPSNTGIDAPTSGAGPAGPYTRQAGFWAYYEIC
TFLKNGATQAWDAPQDVPYAYQGNEWVGYDDVKSFGIKAQWLKENNFGGAMVWAIDLDD
FTGTFCNQGKFPLVNTLKKALGLQSASCTAPAQPVEPIPSPPRSASGNGNSSSGSSSGS
SSGSSSGGSGFCAGKANGLYPVANNRNAFWHCLNGVTYQQNCQAGLVFDTSCDCCNWAA
RGHPFEGKPIPNPLLGLDSTRTGHHHHHH
```

C. Protein A-pig-Chia-V5-His
614 amino acids 67,658 dalton

```
AQHDEAVDNKFNKEQQNAFYEILHLPNLNEEQRNAFIQSLKDDPSQSANLLAEAKKLND
AQAPKVDNKFNKEQQNAFYEILHLPNLNEEQRNAFIQSLKDDPSQSANLLAEAKKLNDA
QAPKVDANSYQLICYFTNWAQYRPGLGSFKPDDIDPCLCTHLVYAFAGMRDNEITTTEG
DDVTFYQSFNGLKNKNSQLKTLLAIGGWNFGTAPFTAMVSAAENRQTFITSVIKFLRQY
GFDGLDFDWEYPGSRGSPSQDKHLFTVLVQEMREAFEQEAKQTKQARLLVTAAVAAGVS
NIQSGYEIPQLSQYLDYIHVMTYDLHASWEGYAGENSPLYKYPTDTGSNAYLNVDYAMN
YWKDNGAPAEKLIVGFPAYGHTFLLSNPSNTDIGAPTSGAGPAGPYTKEAGFWAYYEIC
TFLKNGATQAWDAPQDVPYAYKGNEWVGYDNVKSFNIKAQWLKQNNFGGAMVWAIDLDD
FTGTFCNQGKFPLINTLKDALGLNSTSCTASAQPSEPSSGTGSGSTTGSGSGSSSSGSS
SGGSGYCAGKADGLYPVANNRNAFWHCQNGITYEQYCQTGLVFDTSCQCCNWAARGHPF
EGKPIPNPLLGLDSTRTGHHHHHH
```

D. Protein A-chicken-Chia-V5-His
618 amino acids 67,820 dalton

```
AQHDEAVDNKFNKEQQNAFYEILHLPNLNEEQRNAFIQSLKDDPSQSANLLAEAKKLND
AQAPKVDNKFNKEQQNAFYEILHLPNLNEEQRNAFIQSLKDDPSQSANLLAEAKKLNDA
QAPKVDANSYVLSCYFTNWAQYRPGLGKYMPDNIDPCLCDHLIYAFAGMSNNEITTYEW
NDETLYKSFNGLKNQNGNLKTLLAIGGWNFGTAKFSTMVSTPENRQTFINSVIKFLRQY
QFDGLDIDWEYPGSKGSPSQDKGLFTVLVQEMLAAFEQEAKQVNKPRLMITAAVAAGLS
NIQAGYQIAELGKYLDYFHVMTYDFHGSWDGQTGENSPLYKGPADTGDLIYFNVDYAMN
YWKSNGAPAEKLLVGFPTYGHSYILKNPSDTAVGAPTSGPGPAGPYTRQSGFLAYYEIC
TFLDSGATQAWDAPQDVPYAYKSSEWVGYGNIKSFNIKIDWLKKNNYGGAMVWSLDMDD
FTGTFCKQGKYPLITTLKNALGQQSSSCVPPAQPNPPITAAPSTGSGSGSGSGSGSSGS
NTGSSGGSGFCAGKANGIYADPTNKSKFYNCNNGETFVQSCQAGLVFDSSCSCCNWAAR
GHPFEGKPIPNPLLGLDSTRTGHHHHHH
```

Figure S3. Deduced amino acid sequences and their molecular masses of recombinant proteins expressed in *E. coli.* The amino acid sequences are color coded. Yellow, mature form of truncated form of Protein A; Blue, Chia protein; Green, V5-His sequence.

(a)

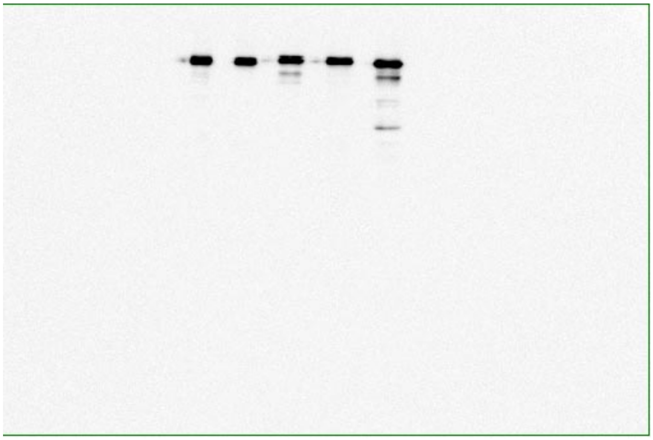

(b)

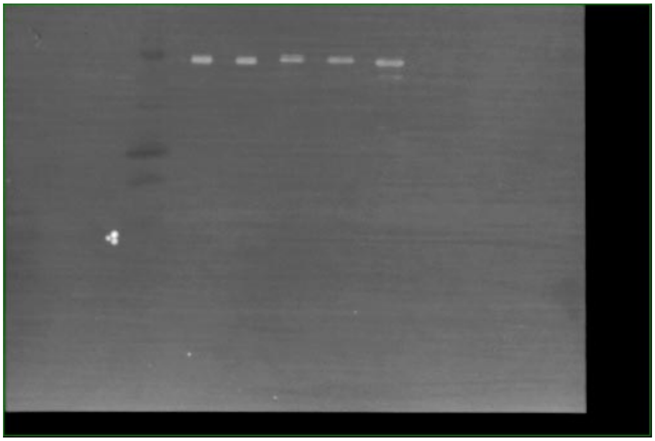

Figure S4. Full-length gel and blots shown in Fig. 3b. (a) Western blotting using anti-V5 antibody. (b) Molecular weight markers of the blot in (a).

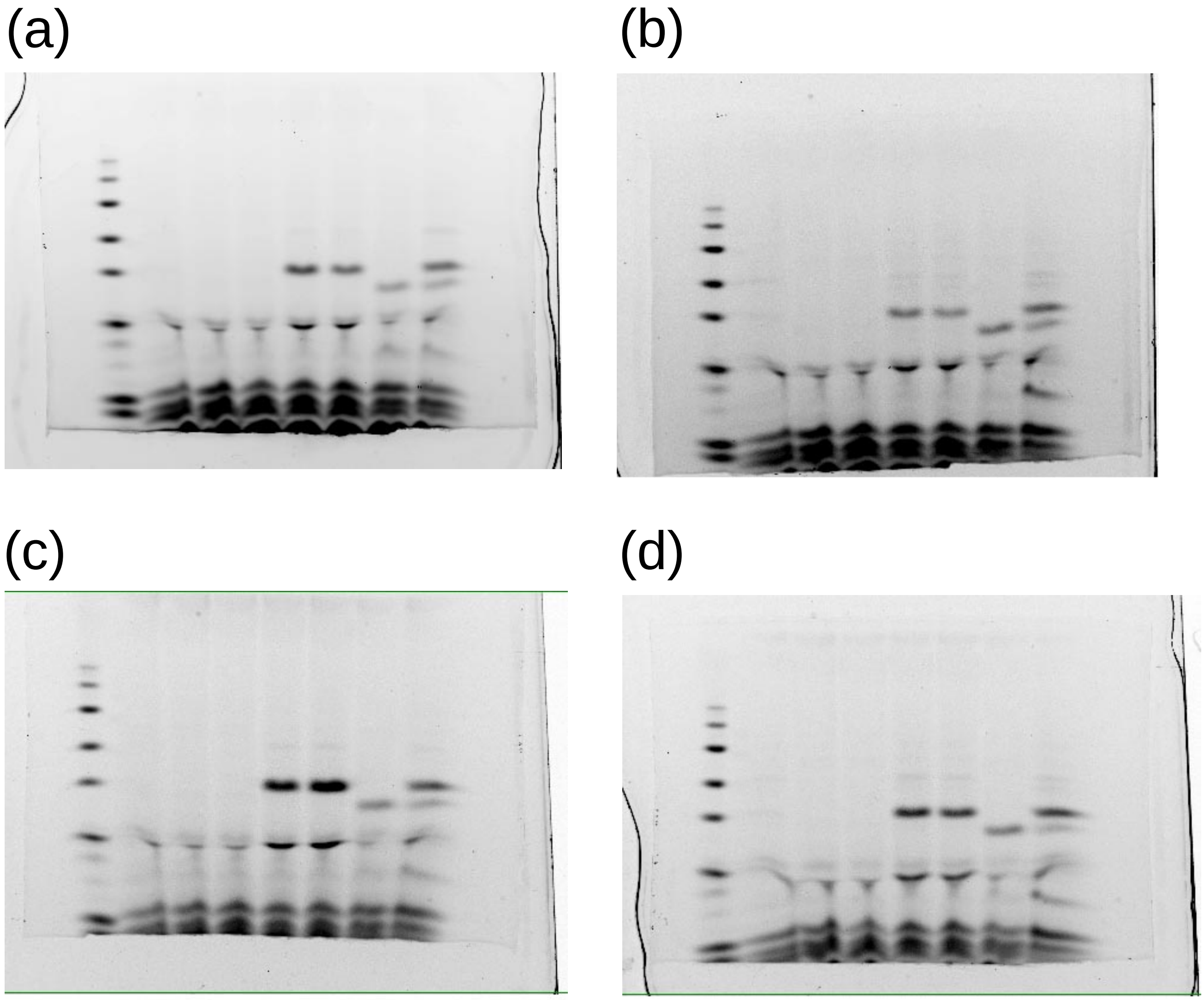

Figure S5. Full-length gel shown in Fig. 5. (a-d) Full-length gel images of FACE methods in Fig. 5a-d.

Table S1. List of qPCR primers.

Dog Chia_Fw: GCTGCTGGTATCTCCAACAT
Dog Chia_Rv: CAGGAGGCATGGAAGTCATAG

Dog_GAPDH_Fw: GAACATCATCCCTGCTTCCA
Dog GAPDH_Rv: CAGGTCAGATCCACAACTGATAC

Dog_Pep_Fw: GCAAACCTATACACTGGCCTA
Dog_Pep_Rv: ATGGGTAGTTGGGTTCATTCTC

Chicken Chia_Fw: GCTGGACATTGACTGGGAATA
Chicken Chia_Rv: CTGAACAAGGACGGTGAAGAG

Chicken GAPDH_Fw: CAACGGATTTGGCCGTATTG
Chicken GAPDH_Rv: GTTCAGATCGATGAAGGGATCA

Chicken Pep_Fw: TGTGGGTGCCCTCTATCTAT
Chicken Pep_Rv: GTAGGCGATGTAGACGGTTTC

Pig_Chia_Fw: TGACTTCACAGGCACTTTCT
Pig_Chia_Rv: CGGTGCAACTTGTGCTATTC

Pig_GAPDH_Fw: GTGACACTCACTCTTCCACTT
Pig_GAPDH_Rv: CCTGTTGCTGTAGCCAAATTC

Pig_Pep_Fw: GGCGGCATCGATTCTTCTTA
Pig_Pep_Rv: GGTGATGCTGTCCAAGGTAAT

Pig_Chit1_Fw: GTCAACTCAGCCATCAGGTT
Pig_Chit1_Rv: CAAGGTCAAGGCCATCAAA

Bovine_Chia_Fw: CACTCACCTGATCTATGCCTTC
Bovine_Chia_Rv: TAGAGAGCCACATCATTCCATTC

Bovine_GAPDH_Fw: GATGCTGGTGCTGAGTATGT
Bovine_GAPDH_Rv: GCAGAAGGTGCAGAGATGAT

Bovine_Pep_Fw: GTCAAGATCCCACTCGTCAAA
Bovine_Pep_Rv: TGCTGCCCAGGTTGTATTT

Bovine_Chit1_Fw: GGCCTTTCTGATTGCCATTTC
Bovine_Chit1_Rv: GTCCACATCCTTGGGTAAGAAG

Mouse_Chia_Fw: TTTTGGCAGTGCATCAATGG
Mouse_Chia_Rv: GCAGCAATTACAGCTGGTATCAA

Mouse_GAPDH_Fw: TGTGTCCGTCGTGGATCTGA
Mouse_GAPDH_Rv: CCTGCTTCACCACCTTCTTGA

Mouse Pep_Fw: TGCCAAGGCATTGTAGACACA
Mouse_Pep_Rv: CTCCTATGGTCTGCAGAAGTTCATT

Mouse_Chit1_Fw: CGGCAGGAACTAAATCTTCCAT
Mouse_Chit1_Rv: TGGGCGTGGCTCAGGTAT

Table S2. Forward and reverse primers used to construct the mammalian and *E. coli*-expression vectors.

EcoRI_Bovine_Chia_Fw:
CGCGGAACCCGAATTCGTACCAGCTGGTATGCTACTTCTCTA

XhoI_Bovine_Chia_Rv:
TTCACCAGCGCTCGAGCTGCCCAGTTGCAGCAGTGACAGCTG

EcoRI_dog Chia_Fw
CATGGAATTCGTACCAGTTGACATGCTACTTCACTA

XhoI_dog Chia_Rv
GTGACCTCGAGGTGCCCAATTGCAGCAATCACAACTG

EcoRI_Pig Chia_Fw
CATGGAATTCGTACCAGCTAATATGCTACTTCACCA

XhoI_Pig Chia_Rv
GTGACCTCGAGGTGCCCAGTTGCAGCACTGACAGCTG

EcoRI_chicken_Chia_Fw
CATGGAATTCGTATGTGCTGTCATGCTATTTCACCA

XhoI_chicken_Chia_Rv
GTGACCTCGAGGTGCCCAGTTGCAGCAGGAACAGCTG
